# Aspirin metabolite sodium salicylate selectively inhibits transcriptional activity of ATF6α and downstream target genes

**DOI:** 10.1038/s41598-017-09500-x

**Published:** 2017-08-23

**Authors:** Fernanda L. B. Mügge, Aristóbolo M. Silva

**Affiliations:** Laboratory of Inflammatory Genes, Instituto de Ciências Biológicas (ICB), Universidade Federal de Minas Gerais (UFMG), Belo Horizonte, MG, Brazil

## Abstract

In response to ER stress, activating transcription factor 6 (ATF6) traffics from ER to Golgi apparatus where it is activated by cleavage before being translocated as transcription factor to the cell nucleus. In this work we describe ATF6α as a newly target of the aspirin metabolite sodium salicylate (NaSal). NaSal treatment of cells induces increases in ATF6α mRNA and protein levels, but these events are not accompanied by ATF6 activation. Conversely, NaSal inhibited ATF6 transactivating activity elicited by various ER stress-inducing stimuli in different cell types. This resulted in reduced expression of a subset of ATF6α target genes. Mechanistically, exposure of cells to NaSal results in ATF6α trapping at the Golgi apparatus, thus preventing nuclear translocation. This study provides evidence that NaSal compound restrains the activity of ATF6α, thereby preventing activation of a specific subset of ER-stress responsive genes implicated in different cellular responses.

## Introduction

Many studies have shown that various non-steroidal anti-inflammatory drugs (NSAIDs) target signaling pathways that are not related to their cyclo-oxygenase (COX) inhibitory mechanism, which was first described for acetylsalicylic acid (ASA, Aspirin)^1^. Growing evidence demonstrate that these compounds exhibit remarkable effects on the unfolded protein response (UPR) (reviewed by ref.^2^), which is triggered by the accumulation of unfolded proteins within the endoplasmic reticulum (ER), the event known as ER stress.

The UPR signaling is elicited within ER upon accumulation of malfolded proteins, resulting in the activation of transmembrane sensors ATF6, IRE1 and PERK. These sensors remain inactive under unstressed conditions, a state which is maintained by the association of their luminal domains to the chaperone GRP78/BiP^3,4^. The dissociation of BiP from their luminal domains had long been taken as the only mechanism responsible for ATF6, PERK and IRE-1 activation. Recent studies, however, have indicated that unfolded proteins can interact directly with the luminal domain of IRE1 and induce its dimerization and further activation^5,6^. A third mechanism has provided evidence that modification of cellular lipid composition sensed by PERK and IRE1 can also lead to UPR activation. In this case, the increased dimerization of their transmembrane domains will eventually promote activation^7,8^.

PERK is activated by acetyl salicylic acid (ASA) and sodium salicylate (NaSal), members of the salicylate class of NSAIDs, in mouse embryonic fibroblasts (MEFs). However, treatment with ASA or NaSal does not affect IRE1-mediated XBP-1 mRNA processing^9^. Nonetheless, salicylates can induce many ER stress related genes, being *Gadd153/Chop* the most upregulated^9–11^, indicating that NaSal affects the expression or activity of ER stress components.

ATF6 is a glycoprotein that has two isoforms (ATF6α and ATF6β), which are ubiquitously expressed^12^. ATF6 activation depends on its translocation from the ER membrane to Golgi apparatus, where its cytosolic N-terminal portion is released by two-step proteolysis regulated by the proteases S1P and S2P^13^. The N-terminal fragments of ATF6α and ATF6β differ in a sequence of eight amino acids, which is responsible for the enhanced transcriptional activity of ATF6α. Additionally, studies performed in MEFs deficient of Atf6α or Atf6β revealed that only the former is required for the transcriptional activation of ER chaperones^14,15^.

Given that ATF6α is implicated in stressful and illness conditions, the investigation of compounds targeting the events downstream of ATF6α ER stress signaling arm is of great interest. Inhibitors of S1P and S2P proteases have been developed, but they also affect the activation of other S1P and S2P targets, such as sterol regulatory element-binding proteins (SREBPs)^16–20^. Drugs that specifically target ATF6 activity, on the other hand, had been unreported until recently. Two studies have demonstrated that a new class of drugs displays specific inhibitory properties on ATF6α activity by trapping of ATF6α in the ER during the organelle stress^21,22^.

Here we show that salicylates also display inhibitory properties on ATF6α activation; however, by a distinct mechanism. NaSal treatment induces an increase in ATF6α mRNA and protein levels with no resulting translocation to the nucleus. Otherwise, NaSal treatment inhibited ATF6α activity by likely blocking its proteolytic processing at the Golgi apparatus during ER stress. Additionally, the activation of ATF6-related transcription factors CREBH and OASIS, which are also cleaved by S1P and S2P proteases at the Golgi apparatus^23–25^, is not inhibited by NaSal. Our findings presented here implicate ATF6α as a novel target for the NaSal biological actions with potential implications in clinical settings.

## Results

### Sodium salicylate elicits the expression of Atf6α in cells

**Figure 1:**
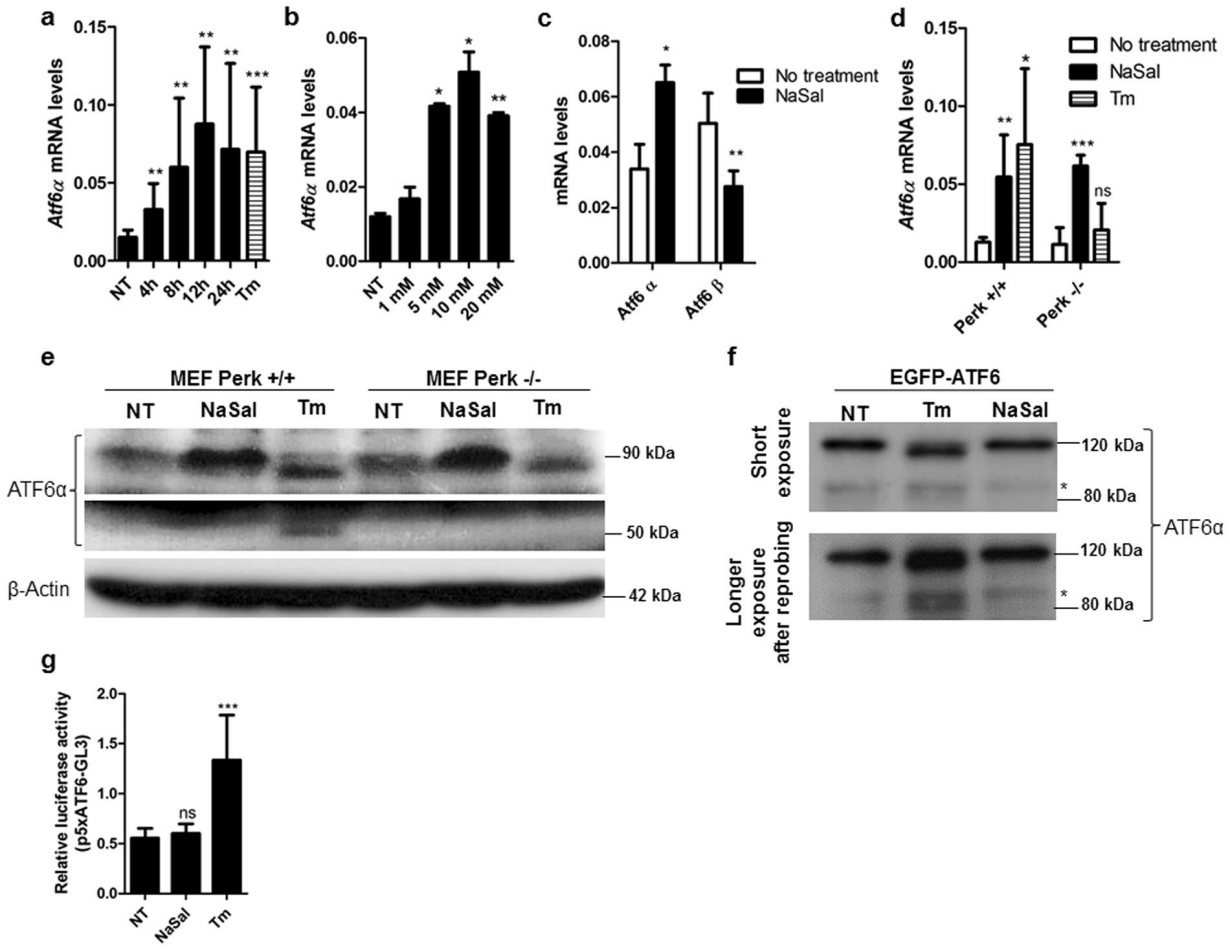
ATF6α expression but not its cleavage is induced in sodium salicylate treated cells. (**a**) MEFs were treated with 20 mM NaSal for the indicated times or 3 µg/mL tunicamycin (Tm) for 8 hours. Total RNA was extracted and RT-qPCR for Atf6α expression was performed after cDNA synthesis. (**b**) MEFs were treated for 12 hours with the indicated concentrations of NaSal and Atf6α expression levels were analysed through RT-qPCR. (**c**) cDNA samples from MEFs treated with 20 mM NaSal for 8 hours were analysed for the expression of Atf6α and Atf6β using specific primers. (**d**) Wild-type and Perk knockout MEFs were left untreated or treated with 20 mM NaSal or 3 µg/mL Tm. Total RNA was extracted and RT-qPCR for Atf6α expression was performed after cDNA synthesis. (**e**) Wild-type and Perk knockout MEFs were left untreated (NT) or treated with 20 mM NaSal or 5 µg/mL Tm for eight hours. Cell lysates were fractionated in 10% polyacrylamid gel, blotted on a PVDF membrane and incubated with anti-ATF6α and anti-β-actin antibodies. (**f**) MEFs were transfected with pEGFP-ATF6. Forty-eight hours after transfection, cells were treated with 20 mM NaSal or 5 µg/mL tunicamycin for eight hours. Cell lysates were blotted and analysed with anti-ATF6α antibody after western blotting. Asterisk indicates a nonspecific band. (**g**) MEFs were transfected with p5xATF6-GL3 and pRL-TK and treated with 20 mM NaSal or 3 µg/mL Tm for 8 hours. Cell lysates were collected and assayed for Firefly and *Renilla* luciferase activities. *^,^ ** and *** indicate statistically significant differences in relation to untreated cells (p < 0.05; p < 0.01; p < 0.001 respectively) as determined by unpaired two-tailed Student’s T-test. ns indicates not significant differences. n = 3 for each experiment and results are plotted as mean ± SD. Western Blots are representative of two independent experiments. Images of full-length western blots can be found in Supplementary Fig. 3.

To assess the expression of Atf6α in response to sodium salicylate, mouse embryonic fibroblasts (MEFs) were treated with NaSal 20 mM at different time points as well as with increasing concentrations of NaSal for 12 hours. The increase in the mRNA levels of *Atf6α* peaked at 12 hours after treatment (Fig. 1a). At this same time interval, the concentration of NaSal capable of inducing an increase in the mRNA levels of Atf6α ranged significantly from 5 to 20 mM (Fig. 1b). Interestingly, the steady state mRNA levels of *Atf6β* in cells were rather decreased upon treatment with NaSal (Fig. 1c). Because the expression of Atf6 can be regulated through PERK^26^, we investigated Atf6α expression in Perk-KO MEFs treated with NaSal and found that while tunicamycin-induced expression of ATF6α was shown to be dependent on PERK, the increase in *Atf6a* mRNA and protein levels induced by NaSal was not affected in the absence of the kinase (Fig. 1d,e). Furthermore, analysis of Atf6α protein levels indicates that the protein accumulates in cells treated with NaSal, but with no evident cleavage of the full ATF6α to its cytosolic portion of 50 kDa. As expected, treatment of cells with tunicamycin led to the cleavage of endogenous ATF6α (Fig. 1e) and EGFP-ATF6 in transfected cells (Fig. 1f), which was accompanied by formation of the full ATF6 doublet into two distinct bands, being the faster migrating the unglycosylated form as predicted^12^ (Fig. 1e,f). In addition, gene reporter studies indicate that NaSal fails to induce any increase in the ATF6-mediated transcriptional activity (Fig. 1g).

### ATF6α-dependent genes are not induced upon sodium salicylate treatment

**Figure 2:**
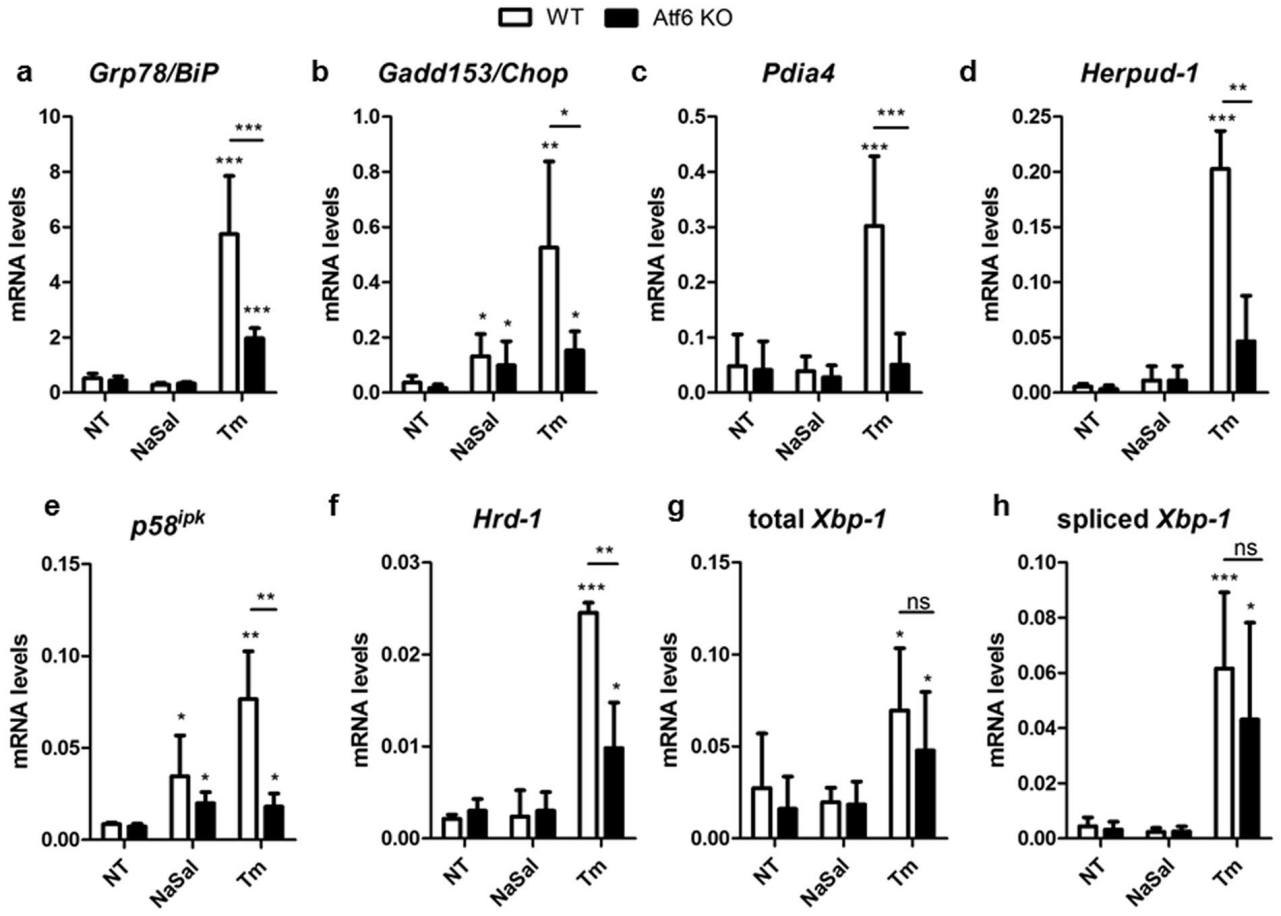
RT-qPCR analysis of ER stress and Atf6α target genes expression in cells treated with sodium salicylate. Wild-type and Atf6α knockout MEFs were treated with 20 mM NaSal or 3 µg/mL tunicamycin for the indicated time. Total RNA was extracted and RT-qPCR analysis of relative mRNA content for BiP/*Grp78* (**a**), CHOP/*Gadd153* (**b**), *Pdia4* (**c**), *Herpud-1* (**d**), *p58*^*ipk*^ (**e**), *Hrd-1* (**f**), total *Xbp-1* (**g**) and spliced *Xbp-1* (**h**) were performed after cDNA synthesis. *^,^ ** or *** indicate statistical significant differences between groups (p < 0.05; p < 0.01 or p < 0.001, respectively) as determined by ANOVA followed by Bonferroni’s test. ns indicates not significant differences. Data were collected from three independent experiments. Results are plotted as mean ± SD.

Intrigued by the result that ATF6 transcriptional activity is not induced in cells treated with NaSal, we next examined the expression of ER stress responsive genes in wild-type and ATF6α knockout MEFs that were treated with NaSal or tunicamycin. The results show that the expression of all chosen target genes, except *Chop/Gadd153* and *p58*^*ipk*^, was not induced by NaSal (Fig. 2a–h). Genes whose expression are partly dependent on ATF6α during tunicamycin-induced ER stress, like *BiP/Grp78* (Fig. 2a), *Pdia4* (Fig. 2c) *Herpud-1* (Fig. 2d), *Hrd-1* (Fig. 2f) and *Xbp-1* (Fig. 2g) showed no significant changes in their steady state mRNA levels in NaSal-treated cells. These genes harbor ATF6-binding elements ERSE, ERSE-II, UPRE and/or UPRE-II^27–29^ in their promoter regions, thereby indicating that Atf6α does not undergo activation in MEFs exposed to NaSal. In addition, the analysis of spliced *Xbp-1* mRNA levels in Fig. 2h confirmed previous observations that IRE1 activation is not induced by NaSal^9^.

### Sodium salicylate inhibits downstream events of ER stress-mediated ATF6α activation

**Figure 3:**
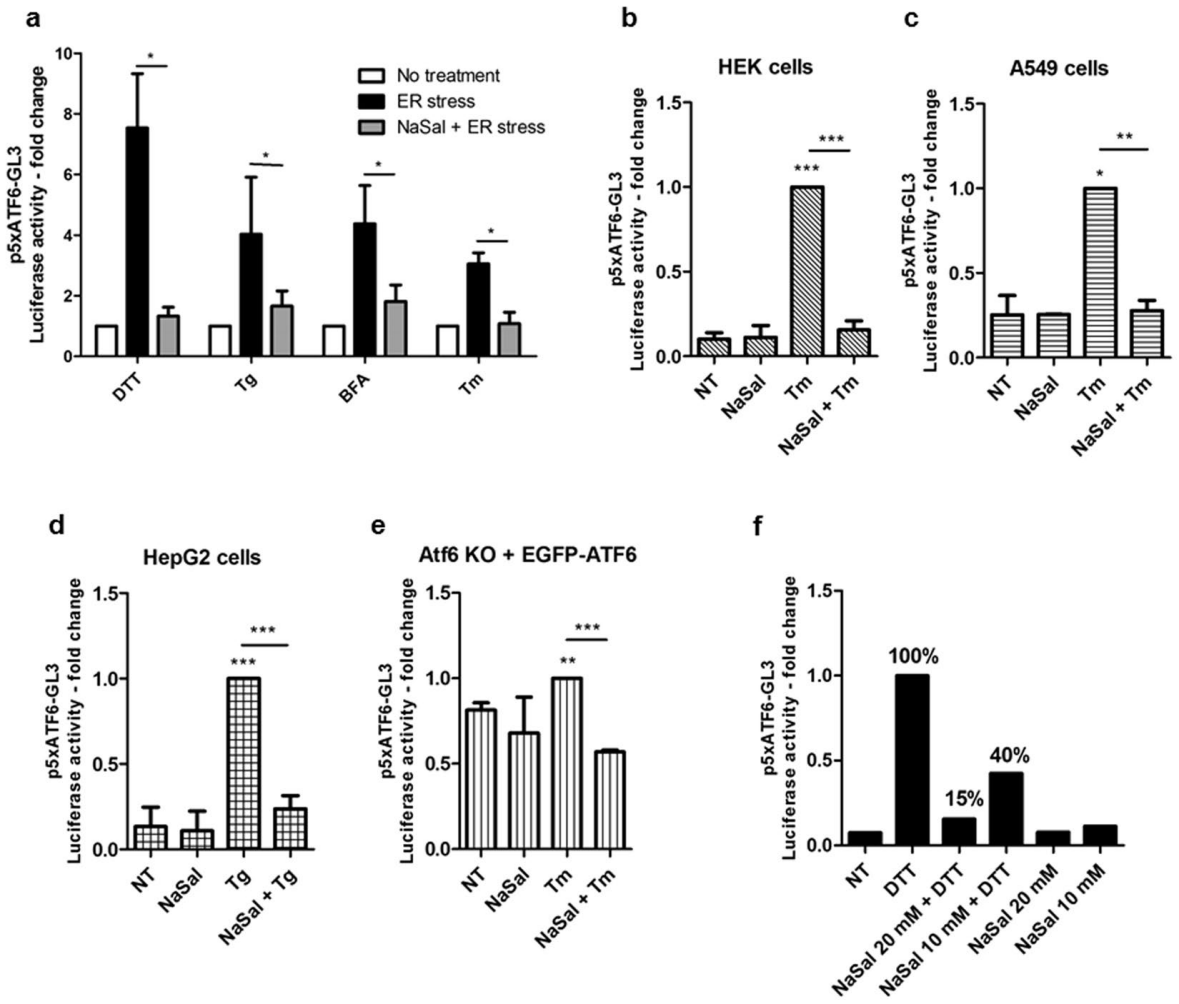
Salicylate inhibits ATF6-mediated ER stress. (**a**) Effect of NaSal on ATF6α transcriptional activity induced by ER stress inducers. Twenty-four hours post-transfection with p5xATF6-GL3 and pRL-TK plasmids, MEFs were left untreated or pretreated for one hour with NaSal 20 mM, followed by treatment or not with tunicamycin (Tm, 3 µg/mL), DTT (1 mM), brefeldin A (BFA, 5 µg/mL) or thapsigargin (Tg, 1 µM) for six hours. Cell lysates were harvested and assayed for Firefly and *Renilla* luciferase activities. Results are presented as the fold increase of luciferase activity in treated cells compared to not treated ones. HEK293 (**b**), A549 (**c**), HepG2 (**d**) cells, and pEGFP-ATF6α-complemented Atf6α knockout MEFs (**e**) were transfected and treated with Tm in the presence or absence of NaSal as described in **a**. As additional control, cells were also left in the presence of the NaSal 20 mM only over the full time of treatment, i.e., seven hours. The medium remained unchanged between treatments. *^,^ ** or *** indicate statistical significant differences between groups (p < 0.05; p < 0.01 or p < 0.001, respectively) as determined by unpaired two-tailed Student’s T-test. n = 3 for each experiment and results are plotted as mean ± SD. (**f**) HEK293 cells transfected with p5xAT6-GL3 and pRL-TK were treated with 20 or 10 mM NaSal and/or 1 mM DTT for identical time interval as in **b**.

We next examined if NaSal would affect ATF6 activation triggered by inducers of ER stress. Then, MEFs were transfected with p5xATF6-GL3 and further pre-treated with 20 mM NaSal for one hour, followed by treatment with DTT, thapsigargin (Tg), brefeldin A (BFA) or tunicamycin (Tm). Figure 3a shows that ATF6α transcriptional activity is significantly induced upon treatment with any of the ER stress-triggering compounds. However, ATF6 activity was dramatically reduced, if not abolished, in cells that were previously exposed to NaSal and further treated with the ER stress compounds. Similar effect was observed in cells that were simultaneously treated with proteasome inhibitor bortezomib (Sup. Figure 1).

We observed that inhibition of transactivating activity of ATF6 by NaSal is not cell type specific, because similar results were obtained in HEK293, A549 and HepG2 cells treated with tunicamycin or thapsigargin (Fig. 3b,c,d). Such an effect was also seen in Atf6α knock-out MEFs transfected with pEGFP-ATF6 that was expressed at similar levels in the different treatment conditions (Fig. 3e; Sup. Figure 2). At 10 mM, NaSal also inhibited ATF6 activation induced by DTT (Fig. 3f).

**Figure 4:**
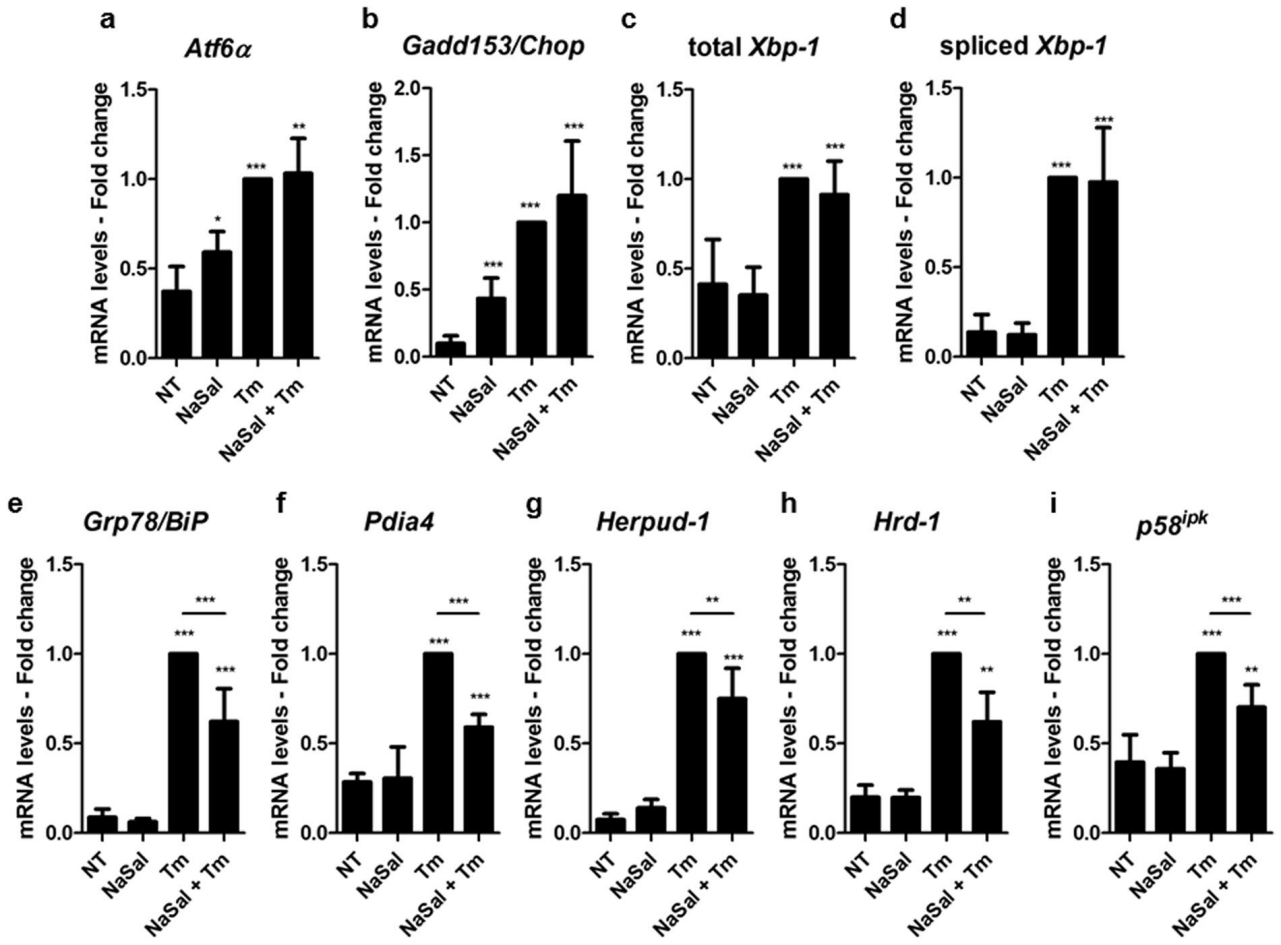
Inhibition of the expression of ATF6α target genes by NaSal. (**a** to **i**) MEFs were left untreated (NT) or treated for one hour with NaSal 20 mM prior to addition of tunicamycin (Tm, 3 µg/mL). After six hours of treatment or not with Tm, total RNA was isolated for cDNA synthesis. Cells were also left in the presence of NaSal 20 mM as described in Fig. 3. Expression of *Atf6α*, *Chop*, total *Xbp-1*, spliced *Xbp-1*, *BiP*, *Pdia4*, *Herpud-1*, *Hrd-1* or *p58*^*ipk*^ was analysed by RT-qPCR. *^,^ ** or *** indicate statistical significant differences between groups (p < 0.05; p < 0.01 or p < 0.001, respectively) as determined by unpaired two-tailed Student’s T-test. n = 3 for each experiment. Results are plotted as mean ± SD.

The analysis of the expression of ATF6α itself and the downstream target genes revealed that the mRNA levels of *Chop/Gadd153*, total and spliced *Xbp-1* remained upregulated in cells that were exposed to NaSal and further treated with Tm (Fig. 4a–d). However, the increase in the mRNA levels of *Grp78/BiP*, *Pdia4*, *Herpud-1, Hrd-1* and *p58*^*ipk*^ upon tunicamycin treatment was significantly impaired in the presence of NaSal (Fig. 4e–i).

### The effects of NaSal on ATF6α activity and its target genes are not affected by the absence of PERK

**Figure 5:**
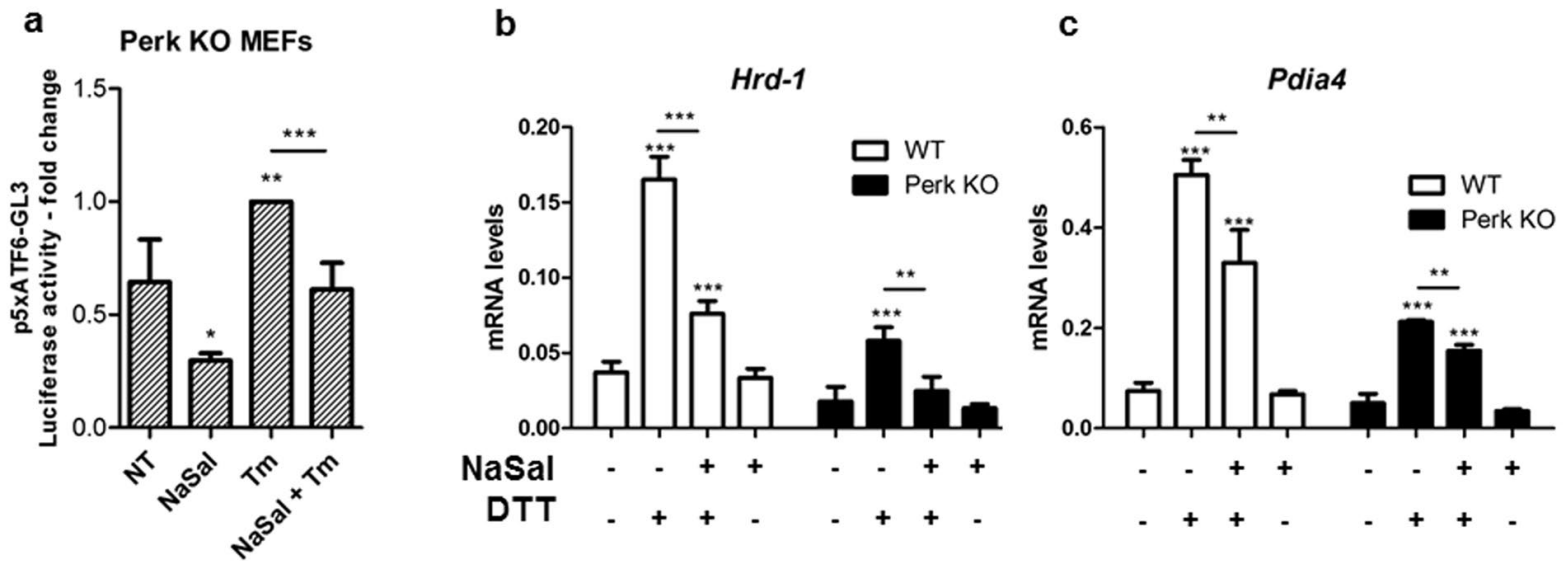
Perk is dispensable for the effects of NaSal on ATF6α activity. (**a**) Effect of NaSal on the Tm-induced ATF6α transcriptional activity in the absence of Perk. Twenty-fours post-transfection with p5xATF6-GL3 and pRL-TK plasmids, Perk KO MEFs were left untreated or pretreated for one hour with NaSal 20 mM, followed by treatment or not with with tunicamycin 3 μg/mL for six hours. Cells were also left in the presence of NaSal 20 mM only for the additional time. (**b** and **c**) Effect of NaSal on DTT-induced expression of ATF6α target genes in the absence of Perk. Wild type and Perk KO MEFs were treated as similar as in Fig. 4, except that DTT (1 mM) was used as the ER stress inducer. Total RNA was isolated for cDNA synthesis and further analyses of *Hrd-1* and *Pdia4* expression by RT-qPCR. *^,^ ** or *** indicate statistical significant differences between groups (p < 0.05; p < 0.01 or p < 0.001, respectively) as determined by unpaired two-tailed Student’s T-test. n = 3 for each experiment and results are plotted as mean ± SD.

Because PERK is implicated in biological events triggered by NaSal^9^ we carried out experiments in MEFs devoid of Perk. The result of gene reporter study (Fig. 5a) indicates that ATF6 transcriptional activity remains significantly inhibited by NaSal even in the absence of Perk. Furthermore, the analysis of the expression of ATF6α target genes *Hrd-1* and *Pdia4* revealed that Perk is dispensable for the inhibitory effects of NaSal (Fig. 5b,c).

### NaSal selectively inhibits the protease-mediated cleavage and nuclear translocation of Atf6α

**Figure 6:**
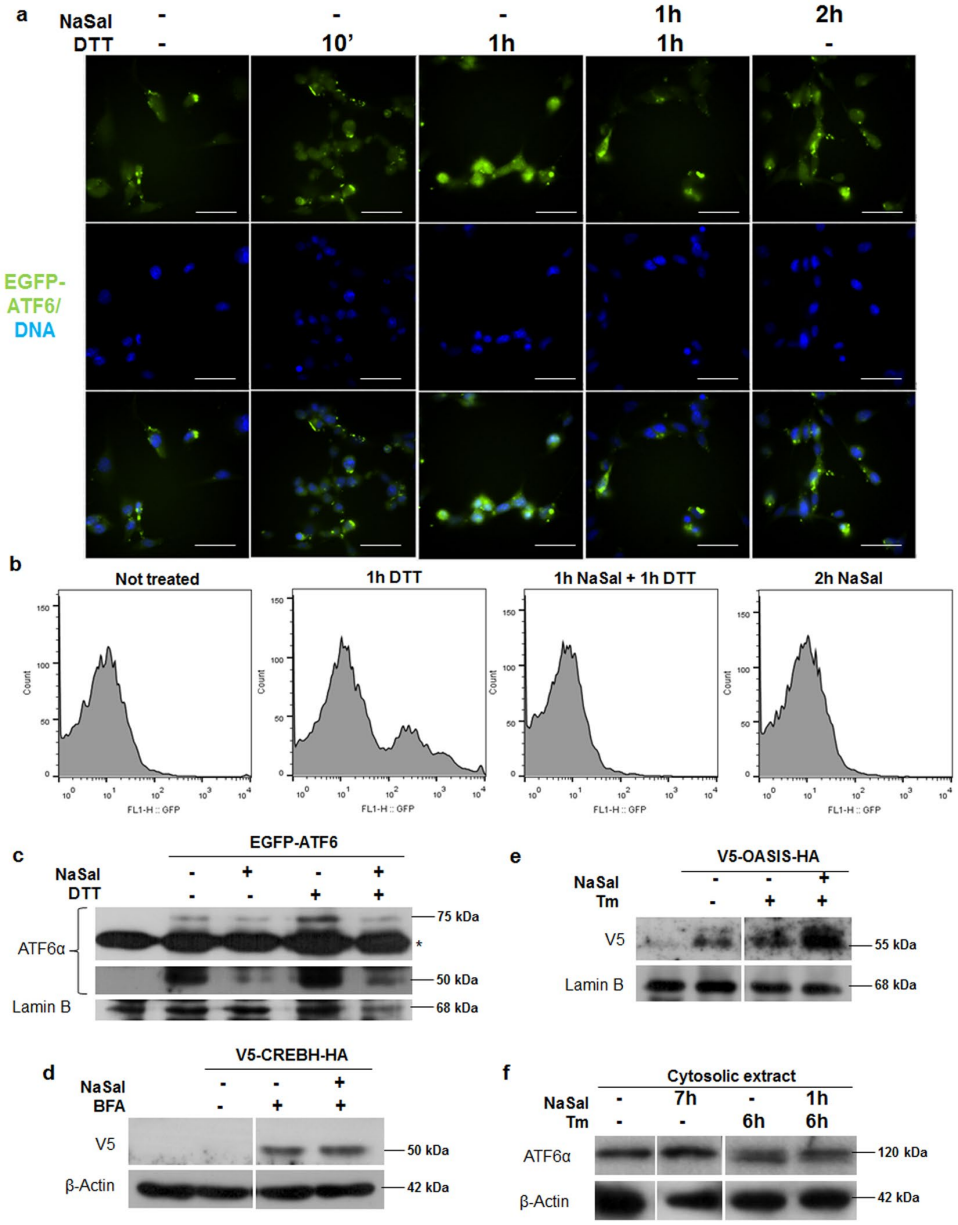
Salicylate inhibits ATF6α translocation to the nucleus. (**a**) MEFs were plated on coverslips and transfected with 1 µg of pCMVshortEGFP-ATF6. Eight hours after transfection cells were treated either with 20 mM NaSal, 1 mM DTT or a combination of both for the indicated times. Cells were then fixed with 4% PFA, counterstained with DAPI and the coverslips were mounted on glass slides. Cells were visualized under 600 x magnification in the fluorescence microscope. White scale bars indicate 50 µm. (**b**) HEK293 cells were transfected with pEGFP-ATF6 treated either with 20 mM NaSal, 1 mM DTT or pre-treated with NaSal and later with DTT. Cells were carefully collected and lysed with nuclei flow cytometry buffer containing 1% Triton X-100. Nuclei obtained after centrifugation were washed and analysed for green fluorescence in a FACSCan flow cytometer. 10.000 events were collected inside the gate determined for nuclei size and granulosity and results were plotted as histograms for green fluorescence intensity versus cell counts. (**c**) Nuclei from HEK293 cells transfected pEGFP-ATF6 and treated with 20 mM NaSal and/or 1 mM DTT were collected following flow cytometry protocol and were lysed for nuclear extract obtaining. Extracts were analysed with anti-ATF6α and anti-lamin B antibodies after western blotting. Asterisk indicates a nonspecific band. (**d**) HEK293 cells were transfected with V5-CREBH-HA for 24 hours where indicated. Cell culture medium was replaced with fresh medium containing 10 µM bortezomib and cells were left untreated, treated with 5 µg/mL brefeldin A (BFA) for six hours or pre-treated with 20 mM NaSal for one hour and later with BFA for six hours. Whole cell extracts were obtained after lysis with RIPA buffer and analysed with anti-V5 and anti-β-actin after western blotting. (**e**) HEK293 cells were transfected with V5-OASIS-HA where indicated. Twenty four hours after transfection, cell medium was replaced with medium containing 10 µM bortezomib, followed by treatment with 5 µg/mL tunicamycin (Tm) for six hours or pre-treated with 20 mM NaSal for one hour and later with Tm for six hours. Cell nuclei were collected and processed as described in (**c**) Samples were analysed using anti-V5 and anti-lamin B antibodies. (**f**) HEK293 cells transfected with pEGFP-ATF6 were treated with 20 mM for the indicated times and/or with 5 µg/mL Tm for six hours. Cytoplasmatic cell extract was collected after cell lysis and nuclei separation and analysed with anti-ATF6α and anti-β-actin antibodies after western blotting. Results are representative of two independent experiments. Images of full-length western blots can be found in Supplementary Fig. 3.

To further investigate whether the cellular localization of Atf6α is affected in cells exposed to NaSal, we performed fluorescence microscopy studies where MEFs were transfected with the pCMVshort-EGFP-ATF6 plasmid^30^ and further treated with DTT or NaSal. The result (Fig. 6a) shows that ATF6α is translocated to the nucleus in cells that were treated with DTT for 1 hour. In cells treated with sodium salicylate and further treated with DTT, however, nuclear translocation of ATF6α is completely abolished. Nonetheless, the protein seems to accumulate at punctate spots near nucleus. Similar observation can be made when cells were treated only with NaSal for 2 hours.

Likewise, flow cytometry studies reveal the absence of ATF6α in the nuclei from HEK293 cells transfected with pEGFP-ATF6 and treated with NaSal plus DTT (Fig. 6b). While the counts of nuclei with higher fluorescence levels increased upon DTT treatment, this is not observed for the nuclei obtained from cells treated with NaSal in the presence or absence of DTT. The N-EGFP-ATF6 cleaved fragment was found in nuclear extracts obtained from tunicamycin treated cells, but not in cells treated with salicylate where the levels of N-EGFP-ATF6α were as similar as in the untreated. This effect is remarkable when the endogenous N-ATF6α (50 kDa) is analysed (Fig. 6c). Taken together, these results indicate that inhibition of ATF6α activity by NaSal is due a likely mechanism that prevents ATF6α from being cleaved, thus hampering its access to the nucleus.

ATF6 and ATF6-related bZIP transcription factors CREBH and OASIS share similar structure and mechanism of cleavage-mediated activation, as they are also ER-located and are activated at the Golgi apparatus by S1P and S2P-mediated cleavage^23,24^. To investigate whether NaSal affects the cleavage of these transcription factors, HEK293 cells were transfected with the encoding plasmids, followed by pre-treatment with NaSal and further treatment with Brefeldin A (CREBH-transfected) or tunicamycin (OASIS-transfected), in the presence of proteasome inhibitor bortezomib. While the cleavage of CREBH induced by Brefeldin A remained unaffected in cells pre-treated with NaSal (Fig. 6d), the cleavage of OASIS induced by tunicamycin seemed to be enhanced in the presence of NaSal (Fig. 6e). These results indicate that NaSal selectively inhibits the proteolysis of ATF6α. Furthermore, the unglycosylated status of ATF6α induced by tunicamycin was found to be mostly unaffected by NaSal pretreatment, indicating that the lack of ATF6α activation is probably not related to its glycosylation levels (Fig. 6f).

**Figure 7:**
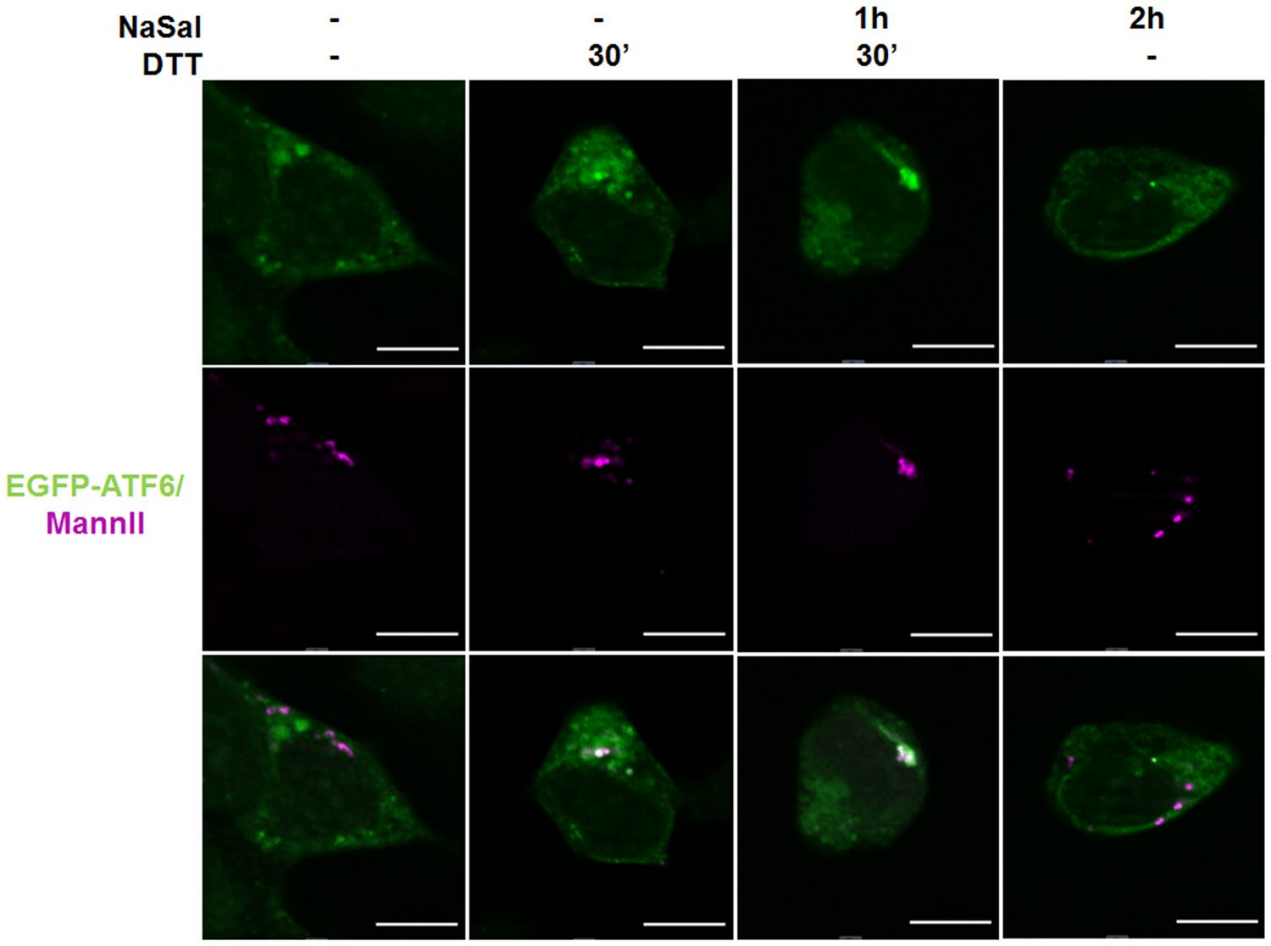
Salicylate treatment traps ATF6α in the Golgi apparatus during ER stress. HEK293 cells were plated on glass coverslips and transfected with 500 ng pCMVshort-EGFP-ATF6 and 20 ng mRuby2-MannII. Twenty four hours after transfection cells were left untreated or treated with 20 mM NaSal and/or 1 mM DTT for the indicated times. Cells were then fixed with 4% PFA and mounted on glass slides. Cell visualization and image capturing were made using an 63 x immersion objective in an Apotome Fluorescence microscope. White scale bars indicate 20 µm. Results are representative of two independent experiments.

Because molecular events at the ER and Golgi apparatus during ER stress precede ATF6α translocation to the nucleus, we determined its subcellular localization in these organelles in cells that were treated with NaSal, in the presence or not of ER stress stimulus. The result (Fig. 7) shows that ATF6α is mostly localized at ER in both untreated and NaSal-treated cells. In cells that were treated with DTT in the absence or presence of NaSal, ATF6α is localized at both ER and Golgi apparatus. This result indicates that in the presence of NaSal ATF6α is retained at Golgi apparatus in ER-stressed cells.

## Discussion

Our findings presented here show that sodium salicylate inhibits the activation of ATF6α transcription factor at the latest events preceding its nuclear translocation triggered by different ER stress inducing stimuli. This led to specific inhibition of the expression of downstream target genes.

Salicylates and other classes of non-steroidal anti-inflammatory drugs have been shown to interfere with the UPR and related pathways^2^. Salicylates can inhibit protein synthesis^31,32^, which was later proved to be dependent on PERK kinase activation coupled to eIF2α phosphorylation^9^. GCN2, another eIF2α kinase, has also been implicated in salicylate-induced effects on ER stress components, as the expression of many ER stress related genes, including ATF6α, was shown to be reduced in GCN2-defficient cells^10^. IRE1, on the other hand, is not activated in salicylate treated cells^9^. NaSal can alleviate ER stress through the reduction of the expression of ATF6, CHOP and BiP on primary human adipocytes exposed to a number of distinct treatment conditions^33^. In hepatic cells, the simultaneous treatment with NaSal and tunicamycin decreased the phosphorylation of PERK and the expression of BiP and CHOP induced by tunicamycin alone^34^.

As the effects of these drugs on ATF6α were not yet fully studied, we investigated the expression and activation of ATF6α on sodium salicylate-treated cells. Our results show that NaSal elicits the expression of *Atf6α* (but not *Atf6β*) in MEFs (Fig. 1a–c), but this is not accompanied by Atf6 activation (Fig. 1g). Also, NaSal treatment promotes Atf6α protein accumulation but not its protease-mediated cleavage processing that occurs during ER stress (Fig. 1e,f). Further investigation revealed that, unlike for the response to tunicamycin treatment^26^, the expression of *Atf6α* induced by NaSal is not impaired in the absence of Perk (Fig. 1d,e). This indicates that an alternate pathway likely mediated through GCN2 kinase is involved in the ATF6α expression in cells treated with NaSal^10^. Consistent with the observation that NaSal treatment induced neither the activation of ATF6-binding site nor the cleavage of full length ATF6α, translocation of EGFP-ATF6 to the nucleus was not observed in NaSal-treated cells (Fig. 6a–c).

As a transcriptional regulator of UPR, ATF6α drives the expression of genes that can increase the ER folding capacity or accelerate the degradation of unfolded proteins. Besides *Gadd153* and *p58*^*ipk*^, whose expression is mostly regulated by the PERK/ATF4 arm of UPR in NaSal-treated cells, none of the other ATF6α target genes studied in this work had their expression induced by salicylate (Fig. 2). This builds up additional evidence that ATF6α activation is not elicited upon NaSal treatment of cells. We also observed that *Xbp1* mRNA processing is not induced by NaSal, confirming previous reported data that IRE1 remains unaffected in the presence of the compound^9^ (Fig. 2h).

In different cell types, ATF6 activation triggered by different ER stress inducing agents was remarkably inhibited by NaSal (Fig. 3a–d). Similar effect was also seen in Atf6α knockout MEFs transfected with EGFP-ATF6, showing that this effect is not exclusive for endogenous ATF6 and is not related to the inhibition of ATF6α mRNA transcription (Fig. 3e). Because the inhibitory effect was also demonstrated in Perk deficient cells (Fig. 5), we ruled out the possibility that the inhibition of ATF6 activation could be a result from salicylate-induced protein synthesis inhibition^9^. Previous studies had demonstrated reduction of the expression of ATF6α, CHOP and BiP on primary human adipocytes exposed to NaSal and ER-stress inducing stimuli for 24 hours^33^. In hepatic cells, the simultaneous treatment with 10 mM NaSal and tunicamycin decreased the phosphorylation of PERK and the expression of BiP and CHOP induced by tunicamycin alone^34^. We found that expressions of *BiP*, *Pdia4*, *Herpud-1*, *Hrd-1* and *p58*^*ipk*^ were significantly but not completely inhibited in cells pre-treated with NaSal (Fig. 4e–i). Since the expression of *Atf6α* itself, *Chop* and spliced *Xbp-1* remained unaffected by NaSal treatment (Fig. 4a–d), these evidence support that the activation of Perk/ATF4 and IRE1/XBP1 pathways induced by tunicamycin remain intact in the presence of NaSal. The absence of PERK, on the other hand, has no impact on the inhibition of ATF6α target genes expression by NaSal (Fig. 5b,c). Regarding these genes, some of the different observations made in other studies could result from variations on the NaSal concentration and time intervals, besides cell types used.

Additional data show that the nuclear translocation of ATF6α induced by ER stress is inhibited by NaSal pre-treatment (Fig. 6a–c). Blockage of ATF6α cleavage by NaSal could be a result of trapping of the protein in the ER, which is the proposed mechanism of action for the newly discovered drugs Ceapins^22^. Nonetheless, subcellular localization of ATF6α in the presence of NaSal indicates accumulation in perinuclear punctae, which co-localize to mannosidase II (Fig. 7), supporting the idea that NaSal traps ATF6α in the Golgi apparatus and inhibits the proteolytic processing mediated by S1P and S2P proteases. ATF6-related b-Zip transcription factors CREBH and OASIS are cleaved by the same proteases in response to brefeldin A and tunicamycin, respectively^35^. However, NaSal did not inhibit the proteolytic processing of CREBH induced by brefeldin A and enhanced OASIS cleavage induced by tunicamycin (Fig. 6d,e), indicating that it does not inhibit S1P and S2P protease activities. We speculate that the selective inhibition of ATF6α cleavage may result from a preceding post-translational modification (PTM) on ATF6α caused by NaSal that would impair its processing by S1P and S2P proteases. S1P and S2P inhibitors, such as AEBSF and Nelfinavir have been previously described, but they do not specifically target ATF6α, and therefore can have uncontrolled effects on other S1P and S2P substrates, like other ATF6-related bZip transcription factors and SREBP transcription factors^16–20^.

Various studies have shown that ATF6 can undergo ubiquitylation^36,37^, and phosphorylation^38–40^, besides undergoing distinct levels of glycosylation^12,41^. In principle, alteration of the glycosylation status of ATF6 by NaSal can be ruled out, as the unglycosylated form of ATF6α induced by tunicamycin remains intact in cells that are pre-exposed to NaSal (Fig. 6f). Aspirin has been shown to inhibit non-enzymatic glycosylation (glycation)^42,43^, but the mechanism is dependent on protein acetylation, which is unlikely to involve the salicylate compound. It is also unlikely that NaSal could somehow provoke phosphorylation of ATF6 as this PTM results not only in an increased ATF6 processing in the Golgi apparatus^38^ but also in nuclear translocation^39^. Finally, we found that the inhibition of ATF6 transactivating activity by NaSal also occurred when cells were simultaneously exposed to proteasome inhibitor bortezomib (Sup. Figure 1). Treatment of cells with proteasome inhibitors has been shown to stabilize N-ATF6^37^. Therefore, if the inhibitory effect of NaSal was a result of accelerated degradation of N-ATF6 by the proteasome, one would expect this effect to be attenuated upon treatment with bortezomib. Gentz *et al*.^10^ showed that NaSal can induce the expression of several ubiquitination genes, including *Sel1l*, which is necessary for the proteasome mediated degradation of full ATF6 and seems to have a positive effect on ATF6 activation^44^. Nonetheless, it could also be possible that NaSal could affect the oligomerization state of ATF6α and therefore impair its activation. The transport to the Golgi apparatus has been shown to occur only for monomeric ATF6α^45^.

Our findings reveal ATF6α as a target for salicylate drugs in various cell types. By the results presented here we demonstrate that (1) NaSal induces ATF6α expression and (2) inhibits the activation of ATF6 during ER stress. While the inhibition of ATF6 activity may result from direct effects of NaSal on the protein activity, the observed effects of NaSal on ATF6α expression make it difficult to rule out indirect effects, that is, being mediated by other cellular components upstream to the transcriptional activation events. Indeed, we were able to previously demonstrate that GCN2 kinase is implicated in mediating the effects of NaSal on the expression of ER stress responsive genes, including ATF6α^10^.

At this point, it is uncertain whether the mechanism by which NaSal inhibits ATF6α activity is direct. However, the evidence shown in Fig. 6c–e, where NaSal selectively inhibits ATF6 cleavage, but fails to inhibit the cleavage of two ATF6-related bZip transcription factors (CREB-H and OASIS) during ER stress, support this idea; especially because they are all targets for S1P and S2P proteases (before being translocated into the nucleus). As this is a very late event that leads to ATF6 activation, it is unlikely that those proteases are inhibited by NaSal. Moreover, NaSal pretreatment of cells did not exhibit inhibitory effects on the other two arms of the unfolded protein response (Fig. 4). It is also possible that in the presence of NaSal, ATF6 may undergo conformational changes that in turn will oligomerize in the Golgi apparatus, and thus prevent it from being cleaved by S1P protease.

It must be highlighted that salicylates are ancient drugs used therapeutically to treat inflammatory disorders as well as to prevent malignancies^46^. Despite a number of reports, it remains unclear how salicylates affect carcinogenesis. Interestingly, activation of ATF6α-Rheb-mTOR pathway promotes survival of quiescent squamous carcinoma cells and subsequent adaptation to chemotherapy^39^. This might be an interesting cellular model to test salicylates as chemopreventive agents in ATF6-mediated chemoresistance of tumor cells.

## Materials and Methods

### Cells, chemicals, plasmids and antibodies

Wild type and ATF6α knockout mouse embryonic fibroblasts (MEFs) were kindly provided by Dr. Randall Kaufman (Sanford-Burnham Institute, La Jolla, CA). Wild type and PERK knockout MEFs were a gift from Dr. David Ron (University of Cambridge, UK). MEFs, A549, HepG2 and HEK293 cells were maintained in Dulbecco’s Modified Eagle Medium supplemented with 10% Fetal Bovine Serum and 1% Penicillin-Streptomycin solution at 37 °C with 5% CO_2_. Sodium salicylate, tunicamycin and brefeldin A were purchased from Sigma. Dithiothreitol (DTT) was from Promega (WI, USA) and thapsigargin from Tocris Biosciences (UK). NaSal was prepared at 1 M in sterile deionized water and then passed through a 0.22 µm filter. Antibodies used in this study include rabbit anti-ATF6α (sc-22799, Santa Cruz Biotechnology, TX, USA), mouse anti-beta actin (Sigma-Aldrich, MO, USA), mouse anti-V5 (Invitrogen, ThermoFisher Scientific, MA, USA), rabbit anti-Lamin B (Santa Cruz Biotechnology), goat anti-rabbit HRP (Cell Signalling Technology, MA, USA) and rabbit anti-mouse HRP (Sigma-Aldrich, MO, USA). Forced expression of EGFP-ATF6 in MEFs was achieved by transfection with pEGFP-ATF6 (purchased from Addgene, #32955^47^) using polyethyleneimine (PEI) (Polysciences, PA, USA). For fluorescence microscopy experiments, cells were transfected with pCMV-short-EGFP-ATF6, a kind gift from Dr. Mori (Kyoto University, Japan)^30^. ATF6α transactivating activity luciferase reporter gene p5xATF6-GL3 (#11976^48^) and the Golgi marker mRuby2-MannII (#55903) were purchased from Addgene. Plasmids for the expression of V5 tagged CREBH and OASIS^49^ were a kind gift from Dr. Peter O’Hare (Imperial College London, UK).

### Real time quantitative PCR (RT-qPCR)

Total RNA was prepared from MEFs using TRIzol reagent (Life Technologies, ThermoFisher Scientific, MA, USA) according to manufacturer’s instructions. First strand cDNA was obtained with MML-V reverse transcriptase (Invitrogen, ThermoFisher Scientific, MA, USA) following the product protocol. The expression of *Atf6α*, *Atf6β, Gadd153/Chop*, *Grp78/Bip*, *Pdia4*, total and spliced *Xbp1*, *Herpud1*, *Hrd1* and *p58*^*ipk*^ was analysed. Primers sequences in the 5′-3′ direction used in this study are: *Atf6α* forward TCGCCTTTTAGTCCGGTTCTT, reverse GGCTCCATAGGTCTGACTCC; *Atf6β* forward TGGAGCAGGATGTCCCGTT, reverse CTGTGGAAAGATGTGAGGACTC; *Herpud1* forward GCAGTTGGAGTGTGAGTCG, reverse TCTGTGGATTCAGCACCCTTT; *Hrd1* forward CGTGTGGACTTTATGGAACGC, reverse CGGGTCAGGATGCTGTGATAAG; *Pdia4* forward TCCCATTGCTGTAGCGAAGAT, reverse GGGGTAGCCACTCACATCAAAT; *p58*^*ipk*^ forward GGCGCTGAGTGTGGAGTAAAT, reverse GCGTGAAACTGTGATAAGGCG; *Gadd153* forward TGCAGTCATGGCAGCTGAGTC, reverse TAGAACTCTGACTGGAATCTG*; Grp78* forward TGGTATTCTCCGAGTGACAGC, reverse AGTCTTCAATGTCCGCATCC*;* total *Xbp1* forward AAGAACACGCTTGGGAATGG, reverse ACTCCCCTTGGCCTCCAC; spliced *Xbp1* forward GAGTCCGCAGCAGGTG, reverse GTGTCAGAGTCCATGGGA. *Rpl32* (forward GCTGCCATCTGTTTTACGG, reverse TGACTGGTGCCTGATGAACT) was chosen by experimental validation as the adequate reference gene among various reference genes that included *Gapdh*, *Hprt*, *Atp5b* and *Cyc1*. Results are presented as mRNA levels calculated as 2 to the power of −ΔCt (where ΔCt is the difference between Cts of the target gene and *Rpl32* reference gene). Where indicated, gene expression in ER stress-treated samples was used as calibrator.

### Cell extracts and immunoblotting

For whole cell extract, cells were washed twice with ice-cold PBS. PBS-EDTA 1 mM was added to the plates and cells were collected in a centrifuge tube. After centrifugation at 600 x g for 5 min, supernatant was discarded and cell pellet was lysed using RIPA buffer (150 mM NaCl; 50 mM Tris-HCl pH 7.4; 1 mM EDTA; 1% Triton X-100; 0.5% Sodium deoxycholate; 0.1% SDS; 1 mM PMSF; 2 mM sodium orthovanadate; 1 µg/mL pepstatin; 2 mg/mL aprotinin; 1 µg/mL leupeptin). After incubation on ice for 30 min, cell extracts were clarified by centrifugation at 12,000 × g for 15 min. Where indicated, cell extracts were fractionated to cytoplasmic and nuclear fractions. Briefly, cells were washed twice with ice cold PBS and a buffer containing 20 mM Hepes pH 7.9; 10 mM KCl; 1 mM EDTA; 1 mM EGTA; 1 mM DTT; 0,25% NP-40 and protease inhibitors as described above was added to the culture plates. After 10 minutes incubation on ice, extracts were centrifuged at 14,000 × g for 1 min. Supernatant containing cytoplasmic fraction was collected into a new tube. For nuclei fractioning, cells were washed twice with ice cold PBS and scrapped with buffer containing 320 mM sucrose; 10 mM Hepes pH 7.4; 5 mM MgCl_2_ and 1% Triton X-100. After incubation on ice for 10 min and centrifugation at 2,000 × g for 5 min the pellet containing cell nuclei was lysed with 20 mM Hepes pH 7.9; 420 mM NaCl; 1 mM EDTA; 1 mM EGTA; 1 mM DTT; 25% glycerol and protease inhibitors, as described, for 20 min on ice. Nuclear extract was collected after centrifugation at 14,000 × g for 5 min. Proteins were quantified with Bradford reagent (Bio-Rad, CA, USA). Forty to 50 µg of cell extracts were fractionated onto SDS-PAGE and transferred to PVDF membranes (Immobilon-P, Millipore, MA, USA). After incubation with primary and secondary antibodies, immunoreactive bands were detected with Enhanced Chemiluminescence reagents (ECL) from Bio-Rad according to manufacturer’s instructions.

### Transient transfection and assay of ATF6 transactivating activity

MEFs, HEK293, HepG2 or A549 cells were plated 24 hours prior to transfection on 24 wells culture plates. Cells were transiently transfected with 400 ng of ATF6 activity reporter p5xATF6-GL3 and 100 ng of pRL-TK *Renilla* reporter using PEI as transfection reagent. Twenty-four hours after transfection, cells were treated either with NaSal, tunicamycin, DTT, brefeldin A or thapsigargin or pretreated for one hour with NaSal and then for additional 6 hours with tunicamycin, DTT, brefeldin A or thapsigargin, without changing the culture medium. Cells were washed once with ice cold PBS and lysed with 200 µL of Passive Lysis Buffer (Promega). Lysates were assayed for firefly and *Renilla* luciferase using Dual-Luciferase reporter assay from Promega. Measurements were performed in a Lumicount-Packard luminometer, and the ratios between firefly and *Renilla* luciferase were calculated.

### Fluorescence microscopy

MEFs were cultured on 13 mm glass coverslips for 24 hours and transfected with 1 µg of pCMVshort-EGFP-ATF6 (provided by Dr. Mori, Japan^30^) using Lipofectamine 2000 (Invitrogen) as recommended by the manufacturer. For the experiments where EGFP-ATF6 and Mannosidase II were transiently co-expressed, transfection of HEK293 cells was performed right after tripsinization and cell resuspension, followed by incubation with DMEM medium containing 500 ng of pCMVshort-EGFP-ATF6, 20 ng of mRuby2-MannII and Lipofectamine 2000. Cells were then plated on glass coverslips. Treatments with NaSal or DTT were performed 24 hours after cell plating or cell transfection, followed by cell fixation with 4% PFA. Cell nuclei were counterstained with DAPI using Vectashield (Vector Laboratories) on the coverslips mounted on glass slides. Fluorescence was visualized in a Nikon Eclipse Ti Fluorescence Microscope. For co-localization experiments, fluorescence was visualized in a Zeiss Apotome Fluorescence Microscope. Both microscopes were used at Centro de Aquisição e Processamento de Imagens (CAPI- ICB/UFMG).

### Flow cytometry

HEK293 cells were transfected with pEGFP-ATF6 and treated with DTT or NaSal 24 hours after transfection. Cells were washed twice with ice cold PBS and scrapped with buffer containing 320 mM sucrose; 10 mM Hepes pH 7.4; 5 mM MgCl_2_ and 1% Triton X-100. After incubation on ice for 10 min, nuclei were centrifuged at 2,000 × g for 5 min and resuspended in 320 mM sucrose; 10 mM Hepes pH 7.4; 5 mM MgCl_2_ without Triton X-100, followed by shearing with a syringe. Nuclei integrity was confirmed by visualization under light microscope using Trypan Blue in a Neubauer chamber. Flow cytometry analysis were performed in a FACSCan equipment.

### Statistical analysis

Results are expressed as mean ± standard deviation (SD) of the indicated number of independent experiments. For statistical comparisons among groups, p values were calculated using two-tailed unpaired Student’s *t*‐test or one-way ANOVA followed by Bonferroni’s multiple comparison test. Differences were considered statistically significant when p < 0.05 (*p < 0.05; **p < 0.01; ***p < 0.001).

### Data availability

All data generated or analysed during this study are included in this published article (and its Supplementary Information files).

## Electronic supplementary material

Supplementary Information
