# Publisher Correction: Aspirin metabolite sodium salicylate selectively inhibits transcriptional activity of ATF6α and downstream target genes

**DOI:** 10.1038/s41598-018-21748-5

**Published:** 2018-03-06

**Authors:** Fernanda L. B. Mügge, Aristóbolo M. Silva

**Affiliations:** Laboratory of Inflammatory Genes, Instituto de Ciências Biológicas (ICB), Universidade Federal de Minas Gerais (UFMG), Belo Horizonte, MG Brazil

Correction to: *Scientific Reports* 10.1038/s41598-017-09500-x, published online 23 August 2017

The original PDF version of this Article contained an error where Figure 6 was truncated during publication. This error has now been corrected in the PDF version of the Article; the HTML version was correct from the time of publication.

