# Supplementary material for: Aspirin metabolite sodium salicylate selectively inhibits transcriptional activity of ATF6α and downstream target genes
Source: Sci Rep. 2017 Aug 23;7:9190. doi: 10.1038/s41598-017-09500-x (PMC5569067; doi:10.1038/s41598-017-09500-x)
Supplement: Supplementary file 1 — Supplementary Information [file 41598_2017_9500_MOESM1_ESM.pdf]

Supplementary information.

Aspirin metabolite sodium salicylate selectively inhibits transcriptional activity of ATF6α and downstream target genes

Fernanda L.B. Mügge and Aristóbolo M. Silva

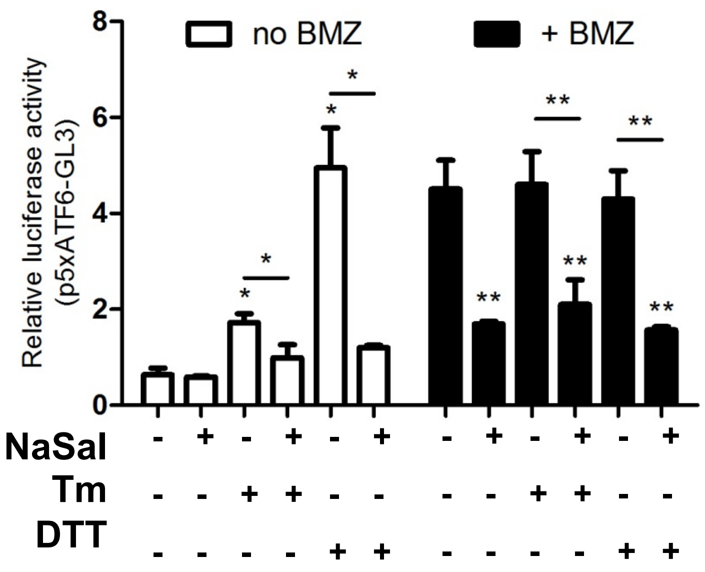

Sup. Fig. 1: Proteasome inhibition does not impact on NaSal effect on ATF6 activation. Twenty-four hours after transfection with p5xATF6-GL3 and pRL-TK MEFs were treated with tunicamycin (Tm, 3 µg/mL), DTT (1 mM) for six hours preceded or not by pre-treatments with 20 mM NaSal for one hour. Where indicated, 10 µM proteasome inhibitor bortezomib (BMZ) was added to cell culture medium. * or ** indicate statistical significant differences between groups ($p<0,05$ or $p<0,01$, respectively) as determined by unpaired two-tailed Student's T-test. n=3 and results are plotted as mean ± SD.

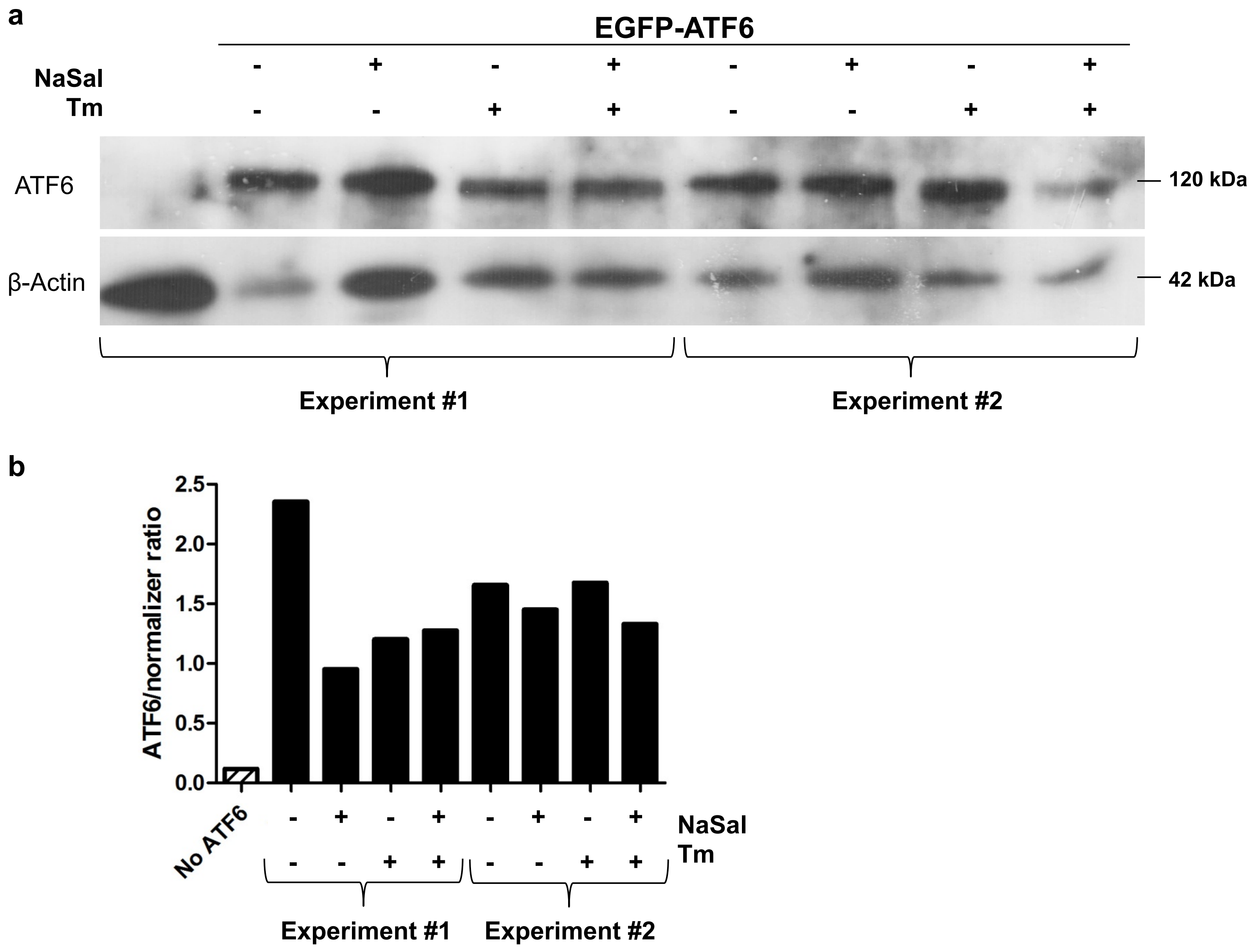

Sup. Fig. 2 (related to Fig.3e): Western-blot analyses of EGFP-ATF6 expression in MEFs lacking ATF6α. a. Five micrograms of the cell extracts analyzed in Fig.3e were fractionated onto 10% SDS-PAGE, transferred to PVDF membranes and immunoprobed with anti-ATF6 and anti-beta-actin antibodies. **b.** Graph shows densitometric analyses of EGFP-ATF6 levels measured by the EGFP-ATF6:beta-actin ratio. Data are expressed as arbitrary units.

Figure 1e

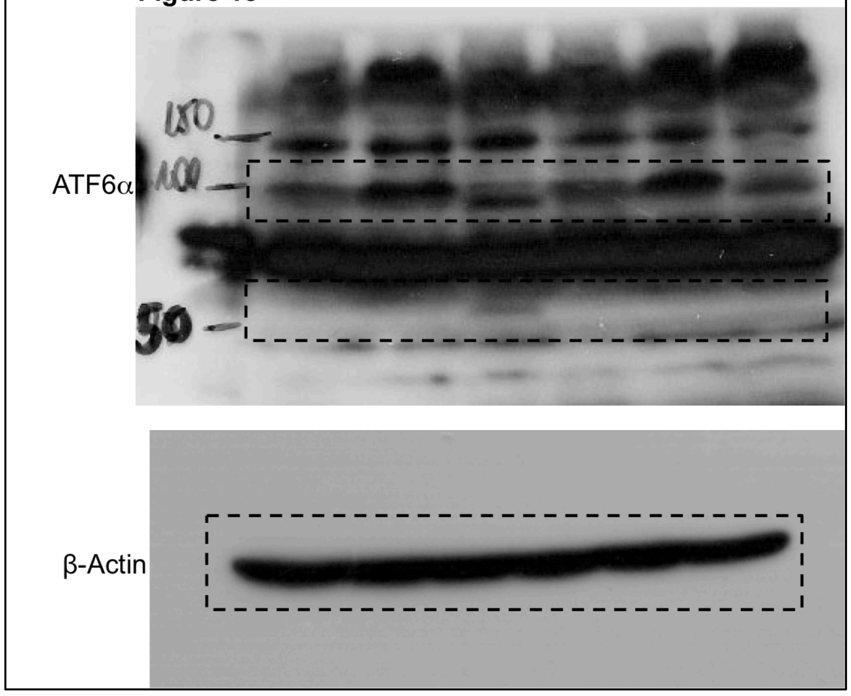

Figure 6c

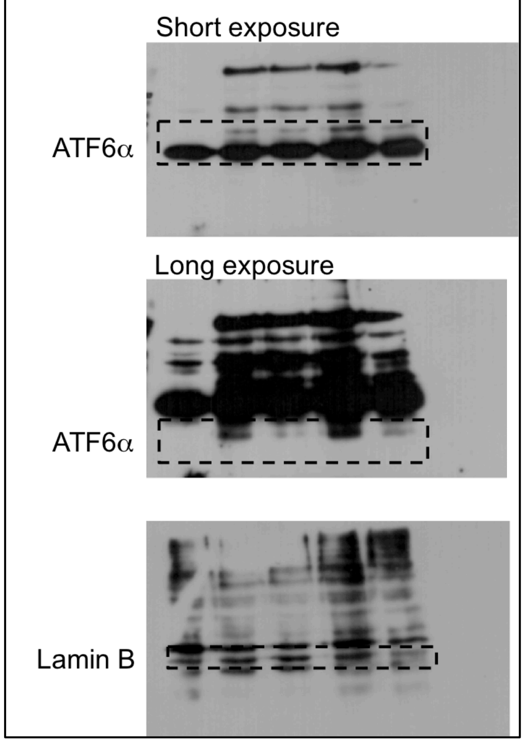

Figure 6d

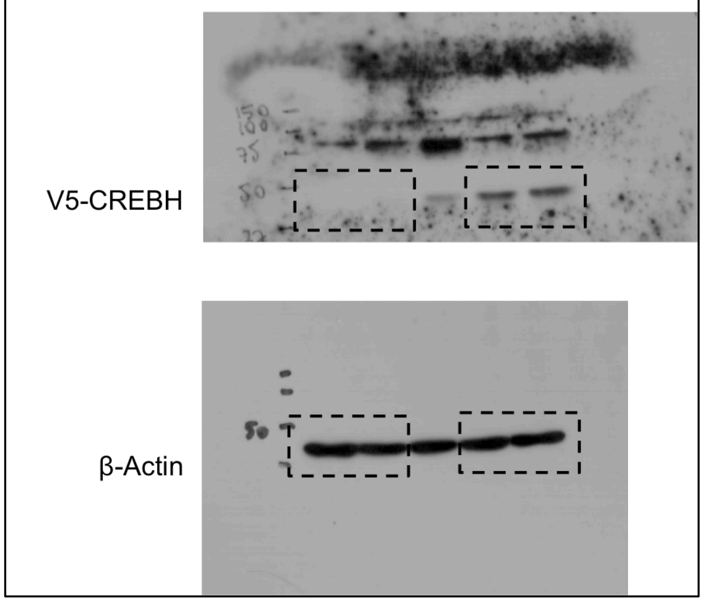

Figure 6e

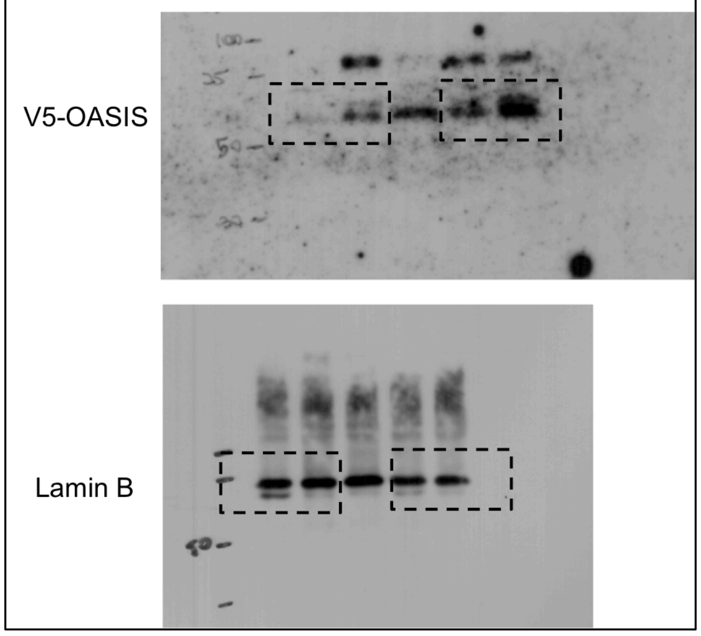

Figure 6f

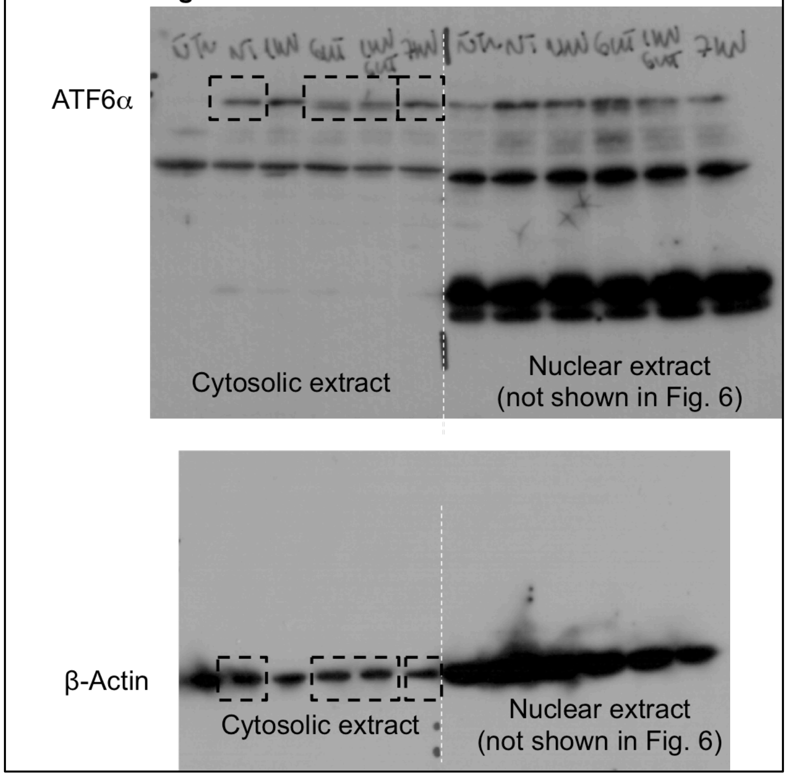

Supplementary Figure 2

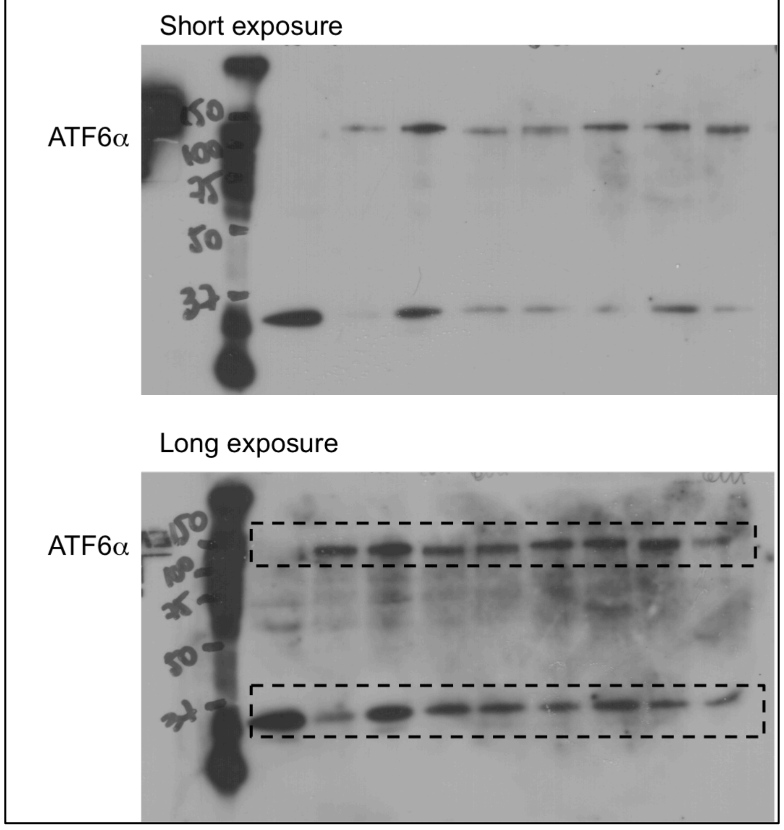

Sup. Fig. 3: Uncropped western-blots related to Figs.1 and 6
